# A Proteomics Analysis to Evaluate Cytotoxicity in NRK-52E Cells Caused by Unmodified Nano-Fe_3_O_4_

**DOI:** 10.1155/2014/754721

**Published:** 2014-08-17

**Authors:** Yi-Reng Lin, Chao-Jen Kuo, Hugo You-Hsien Lin, Chin-Jen Wu, Shih-Shin Liang

**Affiliations:** ^1^Department of Biotechnology, Fooyin University, 151 Jinxue Road, Kaohsiung 83102, Taiwan; ^2^Graduate Institute of Medicine, Kaohsiung Medical University, 100 Shih-Chuan 1st Road, Kaohsiung 80708, Taiwan; ^3^Division of Nephrology, Department of Internal Medicine, Kaohsiung Medical University Hospital, Kaohsiung Medical University, 100 Shih-Chuan 1st Road, Kaohsiung 80708, Taiwan; ^4^Department of Internal Medicine, Kaohsiung Municipal Ta-Tung Hospital, Kaohsiung Medical University, Kaohsiung, Taiwan; ^5^Kaiser Pharmaceutical Co., Ltd., 9 Huan-Gong Road, Tainan 71041, Taiwan; ^6^Department of Biotechnology, College of Life Science, Kaohsiung Medical University, 100 Shih-Chuan 1st Road, Kaohsiung 80708, Taiwan; ^7^Center for Resources, Research and Development, Kaohsiung Medical University, 100 Shih-Chuan 1st Road, Kaohsiung 80708, Taiwan

## Abstract

We synthesized unmodified Fe_3_O_4_ nanoparticles (NPs) with particles size from 10 nm to 100 nm. We cultured NRK-52E cell lines (rat, kidney) and treated with Fe_3_O_4_ NPs to investigate and evaluate the cytotoxicity of NPs for NRK-52E cells. Through global proteomics analysis using dimethyl labeling techniques and liquid phase chromatography coupled with a tandem mass spectrometer (LC-MS/MS), we characterized 435 proteins including the programmed cell death related proteins, ras-related proteins, glutathione related proteins, and the chaperone proteins such as heat shock proteins, serpin H1, protein disulfide-isomerase A4, endoplasmin, and endoplasmic reticulum resident proteins. From the statistical data of identified proteins, we believed that NPs treatment causes cell death and promotes expression of ras-related proteins. In order to avoid apoptosis, NRK-52E cell lines induce a series of protective effects such as glutathione related proteins to reduce reactive oxygen species (ROS), and chaperone proteins to recycle damaged proteins. We suggested that, in the indigenous cellular environment, Fe_3_O_4_ NPs treatment induced an antagonistic effect for cell lines to go to which avoids apoptosis.

## 1. Introduction

In general, nanoparticles (NPs) are a group of particles with diameter size between one nanometer to one hundred nanometers. NPs have properties such as a high specific surface area and have a high potential to be a good catalytic agent, and therefore NPs have been widely used in fields such as antibacterial [1], antimicrobial [2, 3], drug delivery [4], and global use in research [5, 6]. However, having high catalytic potential, NPs were modified with polysaccharide or chitosan to avoid cytotoxicity [2, 3]. Based on the response of toxicity, NPs such as those that use silver and titanium dioxide were evaluated for cytotoxicity, DNA damage, and reactive oxygen species (ROS) [7–9]. Especially in AgNPs, numerous studies showed that in cellular responses, 100 nm AgNPs induced serine/threonine protein kinase (PAK), phosphatase 2A, and mitogen-activated protein kinase (MAPK) pathways, and 20 nm AgNPs induced ROS, SUMOylation, and protein carbonylation [10]. Moreover, published papers increasingly show that proteomics analysis and LC-MS/MS were utilized to demonstrate the effects of NPs, such as quantitative proteomics to evaluate AgNPs exert cellular responses [10], AgNPs treatment directly involved in ROS, metal detoxification according to genomics and proteomics results [11], and the toxic effects and behavior in* Caenorhabditis elegans* [12].

Previous studies reported that* in vitro* experiments and AgNPs exposure directly induced ROS, cell death, apoptosis, and inflammation [13, 14]. In animal model studies, female rats had AgNPs accumulation in kidney regions, especially in the glomerulus [15]. A variety of reports showed evidence that AgNPs treatment has cytotoxicity in proteomics and genomics; however no evaluated studies of other NPs are described with Fe_3_O_4_ or gold nanoparticles.

MS-based quantitative proteomics has been developed at a marvelous rate in the past two decades for biomarker discovery and drug screening. In addition, proteomics provides numerous proteins expression profiles using quantitative techniques to estimate and established the relationships using bioinformatics software. Current quantitative comparisons between specimens both with drug treatment and without treatment or normal and abnormal tissues are beneficial to identify the proteins with upregulation or downregulation and to set up biologic mechanisms and pathways [16, 17]. However in complicated tissue specimens, abundant proteins interfere with the detection of rare proteins. Multiple dimensional separation systems were used to fractionate peptides into different fractions through liquid phase chromatography (LC) to decrease the samples' complication and to increase the amount of protein identification [16, 18].

In this work, we synthesized bare Fe_3_O_4_ NPs and characterized the size of NPs by transmission electron microscope (TEM). After treatment with unmodified Fe_3_O_4_ NPs, we next utilized dimethyl labeling quantitative reagents to label the tryptic peptides of NRK-52E cell lines with treated and untreated Fe_3_O_4_ NPs [19]. In global proteomics research coupled with LC-MS/MS, we demonstrated 435 identified proteins in NRK-52E cell lines by Mascot and simultaneously quantitated 311 proteins through the use of Mascot Distiller bioinformatics software. We classified proteins into chaperone proteins, cell death related and apoptotic proteins, ras-related proteins, and glutathione related proteins. Using STRING for protein-protein interaction [20], we demonstrated the relationships between Fe_3_O_4_ NPs and multifarious proteins.

## 2. Materials and Methods

### 2.1. Chemical Reagents

Sodium acetate, ferric chloride (FeCl_3_), ferrous chloride (FeCl_2_), sodium cyanoborohydride (NaBCNH_3_), and trifluoroacetic acid (TFA) were obtained from Sigma-Aldrich (St. Louis, MO, USA). Acetonitrile (MeCN) was bought from Merck (Seelze, Germany). Ammonium hydrogen carbonate (NH_4_HCO_3_), hydrochloric acid, sodium hydroxide, sodium dodecyl sulphate (SDS), and ethanol were purchased from J. T. Baker (Phillipsburg, NJ, USA). Formaldehyde-D_2_ solution (20% solution in D_2_O) was purchased from Isotec Corp. (Miamisburg, OH, USA), while formaldehyde-H_2_ solution (36.5%–38% in H_2_O), potassium chloride (KCl), sodium chloride (NaCl), sodium dihydrogen phosphate (NaH_2_PO_4_), potassium dihydrogen phosphate (KH_2_PO_4_), dimethyl sulfoxide (DMSO), formic acid (FA, 98%–100%), and iodoacetamide (IAM) were purchased from Sigma (St. Louis, MO, USA). Trypsin was purchased from Promega (Madison, WI, USA). The protein concentration of cell lysate was determined by Bradford assay based on the measurement BSA calibration curve (Thermo, Rockford, IL, USA). Deionized H_2_O with a resistance of 18.2 MΩ was obtained using a Millipore water system.

### 2.2. Fabrication of Fe_3_O_4_ Nanoparticles (NPs) by Hydrothermal Precipitation

The nanoscale magnetic iron oxide particles were prepared by hydrothermal homogeneous coprecipitation [21–23]. Ferric chloride (FeCl_3_, 5.2 g) and ferrous chloride (FeCl_2_, 2.0 g) were dissolved with sonication in 50 mL clean glassware containing aqueous hydrochloric acid (2 M, 25 mL) at 50°C. Subsequently, the mixture was degassed continuously using a nitrogen gas cylinder. Sodium hydroxide (NaOH 150 mL, 2 M) was slowly injected drop by drop into the glassware. The color of the solution transformed from yellow to orange, and finally the color shifted to black through the NaOH dripping down. After incubation at 50°C for one hour, the supernatant was removed through a strong magnet absorbing NPs. The generated NPs were then washed with deionized water and removed by a centrifugal tube with 1,800 rpm for two minutes. Then finally the NPs were rinsed with 0.5 M HCl, deionized water and ethanol sequentially.

### 2.3. Characterization of Fe_3_O_4_ by Transmission Electron Microscope (TEM)

The size of the synthetic Fe_3_O_4_ NPs was characterized by a JEOL JEM 1200-EX transmission electron microscope (TEM) with the accelerating voltage 80 keV. One *μ*L of the nanoparticles solution was dropped on a cupric grid with stabilized carbon, and subsequently the surplus solution evaporated in the atmosphere.

### 2.4. NRK-52E Cell Culture, the Conditions of Fe_3_O_4_ NPs Treatment, and Protein Concentration

The cells, NRK-52E (CRL-1571; American Type Culture Collection, Manassas, VA), were cultured in Dulbecco's modified Eagle's medium (DMEM, Sigma-Aldrich) and supplemented with 5% fetal bovine serum (FBS) and 1% penicillin (Gibco, Grand Island, NY, USA). Cells were cultured in a 100 mm dish with a 5% CO_2_ incubator at 37°C. Until cells reached 80% confluence, they were cultured with the absence of serum medium for 24 hours. After starvation cells were treated with 1 ng Fe_3_O_4_ NPs in each dish for 24 hours, and comparison groups were treated with dimethyl sulfoxide (DMSO). After being washed three times with phosphate-buffered saline (PBS), the cells were lysed with modified RIPA buffer containing 1% NP-40, 0.1% SDS, 150 mM NaCl, 50 mM Tris-HCl, and 1 tablet/10 mL of Roche minicomplete protease inhibitor cocktail at pH 7.5. Finally, the protein concentration of NRK-52E cell lysate was determined using the Bradford assay.

### 2.5. Tryptic Digestion, Dimethyl Labeling, Desalting, and Fractionation

Samples of lysated protein solution 60 *μ*L containing 100 *μ*g of total proteins treated with Fe_3_O_4_ NPs and DMSO were reduced by reaction with 0.7 *μ*L of 1 M DTT and 9.3 *μ*L of 7.5% SDS at 95°C for 5 minutes. The sample bottles were put on ice and then for alkylation were treated with 8 *μ*L of 50 mM IAM at room temperature for 30 minutes in the dark with agitation. Furthermore, sample proteins were precipitated by 52 *μ*L of 50% trichloroacetic acid (TCA) with incubation on ice for 15 minutes. By centrifugation to remove the supernatant, the protein pellets were washed in different solutions according to priority with 150 *μ*L of 10% TCA, 250 *μ*L of deionized H_2_O, 250 *μ*L of acetone, and two times with 250 *μ*L of deionized H_2_O. After removing the supernatant, the protein pellets were digested in 2 *μ*g of trypsin in 200 *μ*L 50 mM NH_4_HCO_3_ (pH 8.5). An additional 2 *μ*g of trypsin was added after 4 hours of tryptic digestion, and then the samples were digested for 14 hours at 37°C. Eventually the tryptic digestion samples were dried by vacuum centrifugation to remove the digested buffer. For labeling procedures, the lyophilized samples were redissolved in 180 *μ*L of 100 mM sodium acetate buffer at pH 5.5. Simultaneously the treated NPs samples were labeled using 10 *μ*L of 4% formaldehyde-D_2_ and then those samples treated DMSO were labeled using 10 *μ*L of 4% formaldehyde-H_2_. Both of the samples were vortexed for 5 minutes, followed by treatment with 10 *μ*L of 600 mM sodium cyanoborohydride for 1 hour. Finally, the labeled solutions were combined after adjusting to pH 2-3 using 10% TFA/H_2_O for desalting by a homemade C18 cartridge desalting kit. After vacuum centrifugation drying, the triplicate combined samples were fractionated with a hydrophilic interaction chromatography (HILIC) separated system. HILIC system was performed using an L-7100 pump system (Hitachi, Tokyo, Japan) connecting a TSK gel Amide-80 HILIC column (2.0 mm × 150 mm, 3 *μ*m particle size; Tosoh Biosciences, Tokyo, Japan) with a flow rate of 200 *μ*L/min. The mobile phase system was used for gradient elution: solvent (A) was 0.1% TFA in water; solvent (B) was 0.1% TFA in 100% MeCN. The eluted fraction from the homemade C18 cartridge was dissolved in 25 *μ*L of solution containing 85% MeCN and 0.1% TFA and then injected into the 20 *μ*L sample loop. Furthermore, the gradient was performed as follows: 95% (B) for 2 minutes; 95%~60% (B) for 32 minutes; 60%~5% (B) for 10 minutes; 5% (B) for 5 minutes; 5%~95% (B) for 1 minute; and 95% (B) for 10 minutes. Each fraction contained 1.2 mL buffer in the total of 10 collected fractions, and finally every fraction was dried in a vacuum centrifuged system.

### 2.6. Protein Identification by LC-MS/MS Analysis

The vacuum dried lyophilized samples were redissolved in 10 *μ*L of 0.1% FA in H_2_O and analyzed using a Thermo LTQ Orbitrap XL (Thermo Fisher Scientific, San Jose, CA). A total of 10 *μ*L of sample was injected onto a C18 capillary pretrapped column (0.3 mm × 5 mm, 5 *μ*m particle size, Agilent Zorbax XDB); HPLC loading pump and separation was performed by a C18 column (i.d. 75 *μ*m × 150 mm, 3 *μ*m particle size, Micro Tech, Fontana, CA). A LC-MS/MS separation was performed using an Agilent 1200 series Nanoflow pump (Agilent Technologies, Santa Clara, CA). The flow rate of loading pump was set at 5 *μ*L/min, and the gradient pump was set at 300 nL/min. The mobile phases consisted of (A) 0.1% FA in water and (B) 0.1% FA in 100% MeCN. The linear gradient was as follows: 2% (B) in 2 minutes, 2%~40% (B) in 40 minutes, 40%~95% (B) in 8 minutes, 95% (B) in 2 minutes, 95%~2% (B) in 1 minute, and 2% (B) in 7 minutes. The digested peptides were detected by a voltage of 1.8 kV in the positive detection ion mode. The separated system with full scanning mode conditions was set at *m*/*z* 400–1600 Da with resolution = 30,000. The data-dependent mode was set according to a priority to select peptides which were detected with 5 high-intensity signals in the MS mode and transferred into collision chamber for fragmentation with a collision energy of 35 eV. The fragmented ions were analyzed and detected at second MS analyzer with a mass range of *m*/*z* 100–2000 Da. To exclude ions with similar *m*/*z* and avoid interferences, the data-dependent mode was also set a repeat duration of 30 seconds.

### 2.7. Mascot Database Search and Mascot Distiller Quantitation

The software Xcalibur (version 2.0.7, Thermo Scientific Inc., San Jose, CA) was utilized to control Orbitrap XL and to acquire the MS and fragmentation data. The raw data including MS and MS/MS spectra were converted to a suitable file type by the Mascot Distiller software (version 2.5.1.0 (64 bits), Matrix Science Ltd., London, UK) to perform protein identification and quantitation. The parameters of the Mascot Distiller were set as follows: “Orbitrap_res_MS2” (default parameter setting) for peak list transformation; “Rattus” for the taxonomy in the Swiss-Prot databank of the Mascot search engine; zero allowable missed cleavages for tryptic digestion; dimethylation (MD) for quantitation; fixed modification was selected carbamidomethyl for cysteine modification; peptide tolerance of 10 ppm with precursor ions; and 0.8 Da tolerance for MS/MS. Peptides charge was selected when they had charges of 1^+^, 2^+^, and 3^+^, and the instrument was set to “ESI-trap.” Finally, the protein quantitative result was listed by the heavy-labeled/light-labeled (D/H) ratios from the Mascot Distiller.

## 3. Results and Discussion

### 3.1. Fe_3_O_4_ NPs Synthesis and Characterization

**Figure 1:**
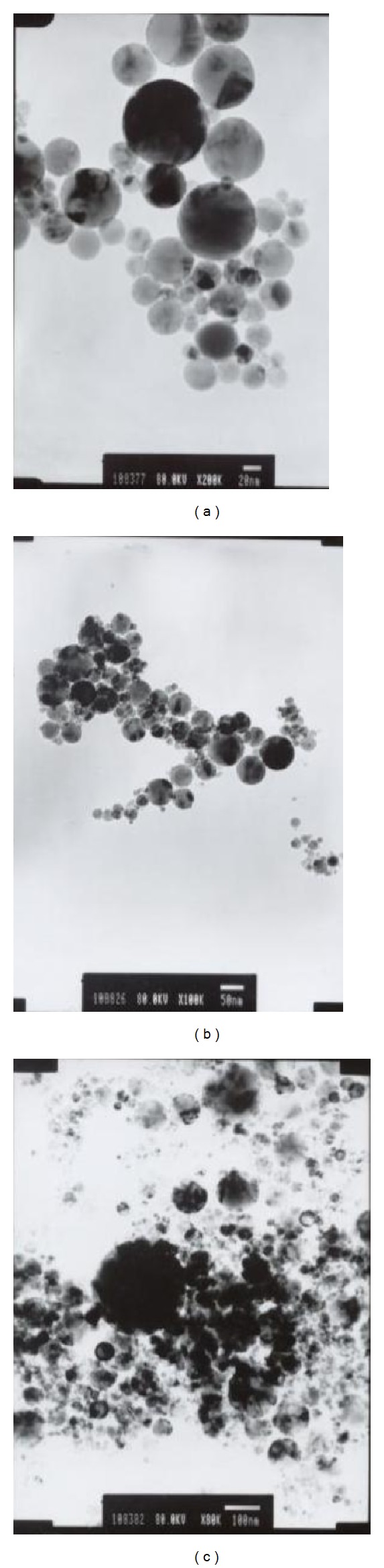
TEM images of Fe_3_O_4_ NPs with different sizes as follows: (a) 20 nm, (b) 50 nm, and (c) 100 nm scale bars.

Bare and unmodified Fe_3_O_4_ NPs were synthesized by hydrothermal precipitation and NPs were fabricated by chemical method to have a uniform particle size. In Figure 1, TEM images showed that we took three photos of Fe_3_O_4_ NPs with 20 nm, 50 nm, and 100 nm scale bars. It is considered that NPs with a higher surface area are easier to aggregate with other NPs; therefore through the aggregation in Fe_3_O_4_ NPs there were Fe_3_O_4_ particles generated above 100 nm if no additional protective agent was modified onto Fe_3_O_4_ (Figure 1(c)). We considered that Fe_3_O_4_ NPs with dispersive diameter sizes are suitable for cytotoxicity evaluation due to the fact that NPs found in the environment have random sizes.

### 3.2. Schematic Representation of Samples Pretreatments, Fractionation, Protein Identification, and Protein Quantitation

**Figure 2:**
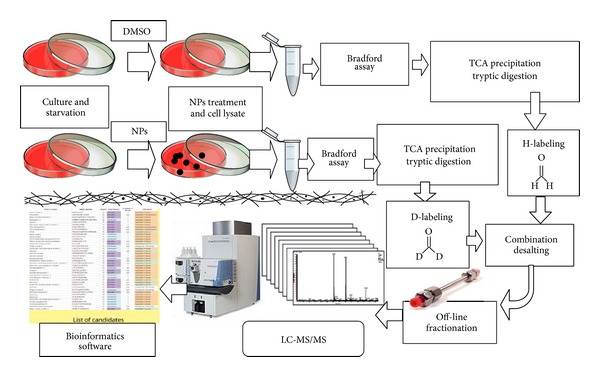
Simplified flow chart of sample pretreatment, fractionation, and analysis using LC-MS/MS.

For protein quantitation, the procedures and using reagents including formaldehyde-H_2_, formaldehyde-D_2_, and sodium cyanoborohydride (NaBCNH_3_) were obeyed as following the previous literature [19]. By utilization of dimethyl labeling, the differences of protein expressions in treatment with and without NPs was enabled to be determined. In the mass spectrum, the proteins of NRK-52E treated NPs were labeled as formaldehyde-D_2_ and proteins of untreated NPs were labeled as formaldehyde-H_2_. In the same sequential peptides, however, different treatments had a mass difference 4 Da or 8 Da depending on the use of the labeling agent. Off-line HILIC fractionation was utilized to decrease the complication of the mixed samples [16, 18]. Through LC-MS/MS, the proteins identification and quantitative ratios of D/H peptides were identified and calculated using the bioinformatic Mascot Distiller software to finally generate a protein list. The schematic flow chart of the process is shown in Figure 2 from sample pretreatment and protein identification to protein quantitation.

### 3.3. Quantitative Results of NRK-52E Proteins Associated with Fe_3_O_4_ NPs

**Figure 3:**
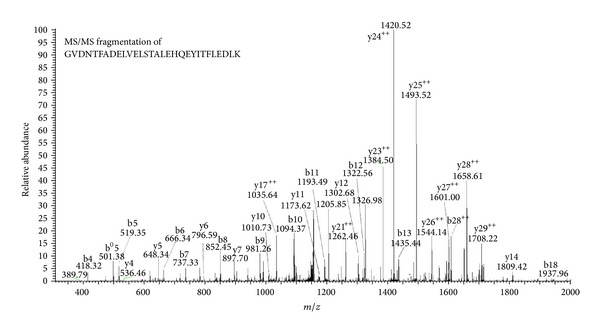
MS/MS spectrum of the peptide sequence GVDNTFADELVELSTALEHQEYITFLEDLK (*m*/*z* 1168.60, 3^+^), which is a peptide of complement component 1 Q subcomponent-binding protein (C1qBP).

**Figure 4:**
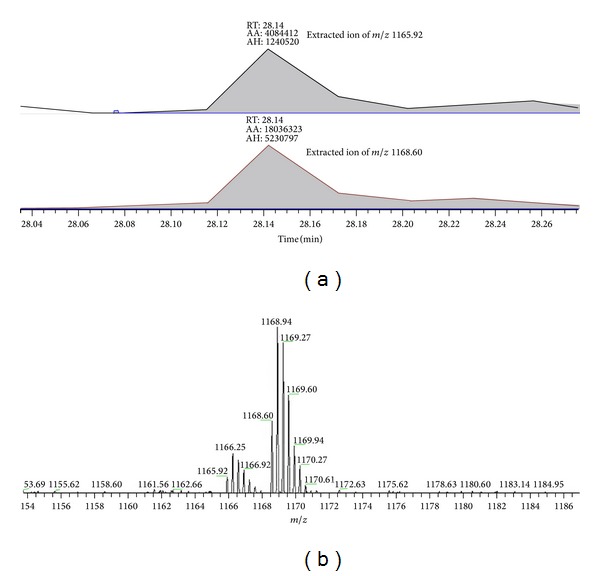
(a) Extracted ions of *m*/*z* 1165.92 (H-labeled) and *m*/*z* 1165.92 (D-labeled) showing signals of peak area (AA) and peak height (AH). (b) MS spectrum of GVDNTFADELVELSTALEHQEYITFLEDLK which are labeled formaldehyde-H_2_ and formaldehyde-D_2_. These two labeled peptides are shown in a coeluted isotopic pattern.

From the statistical results we identified 435 proteins, of which 311 proteins have a D/H ratio and 124 proteins which we were unable to specific values. For example, complement component 1 Q subcomponent-binding protein (C1qBP) was identified with an arithmetic average D/H ratio of 4.23.  Figure 3 showed the peptide belonging to C1qBP having sequences GVDNTFADELVELSTALEHQEYITFLEDLK which was fragmentized by collision induced dissociation (CID), and its MS/MS spectrum was illustrated by y-ions and b-ions. From a quantitative standpoint in support of the Mascot Distiller in bioinformatics software, the result of protein list showed that the D/H ratio belonging to GVDNTFADELVELSTALEHQEYITFLEDLK peptide was 4.23. Simultaneously the raw data from LC-MS/MS that we extracted from the labeled peptide of C1qBP by *m*/*z* 1165.92 (3^+^, H-labeled) and 1168.60 (3^+^, D-labeled) presented signals of peak area and peak height (Figure 4(a)) and showed the isotope pattern of H-labeled peptide and D-labeled peptide (Figure 4(b)) which demonstrated the changes in different labeling. The statistical results showed that peak area was 4.42-fold in D/H ratio and peak height was 4.22-fold; the statistical data was calculated and shown in Table 1.

### 3.4. Differentially Expressed Proteins of NRK-52E by Treatment with Fe_3_O_4_ NPs

In this study, we used Fe_3_O_4_ NPs to treat NRK-52E and used bioinformatics software to generate proteins identification and proteins quantitation with a protein list (the protein list is shown in the electronic supplementary information available online in the Supplementary Materials at http://dx.doi.org/10.1155/2014/754721). In the protein list, we observed that when Fe_3_O_4_ NPs were treated, ras-related proteins were expressed including ras-related protein Rab-7L1 (RAB7L_RAT, 3.2-folds), ras-related protein Rab-1A (RAB1A_RAT, 2.3-folds), ras-related protein Rab-2A (RAB2A_RAT, 1.6-folds), ras-related C3 botulinum toxin substrate 1 (RAC1_RAT, 1.2-folds), ras-related protein Rap-1A (RAP1A_RAT), and ras-related protein Rab-7a (RAB7A_RAT). According to previous studies, ras-related proteins were associated with cancer cell metastasis [24, 25], and ras-related C3 botulinum toxin substrate 1 was associated with differential roles such as cell proliferation which could be inhibited by miR-101 [26] and signal transduction to upregulate in esophageal squamous cell carcinoma and esophageal adenocarcinoma [27].

We also identified related proteins in cell death and apoptosis, such as apoptotic protease-activating factor 1 (APAF_RAT, 780.9-folds), programmed cell death protein 10 (PDC10_RAT, 233.2-folds), galectin-1 (LEG1_RAT, 1.75-folds), and programmed cell death 6-interacting protein (PDC6I_RAT). Apoptotic protease-activating factor 1 activated procaspase-9 and modulated cellular apoptosis [28]. However, programmed cell death protein 10 promotes cell proliferation and protects malignant T cells from apoptosis [29]. The galectin family played different roles in cell apoptosis such as galectin-7 enhancing apoptosis, but galectin-1 has the opposite effect [30]. In accordance with previous studies and the proteins quantitative list, we considered that the treatment of Fe_3_O_4_ NPs caused an antagonistic effect. Fe_3_O_4_ NPs induced expression of ras-related proteins and induced programmed cell death protein 10, galectin 1 to promote cell metastasis, proliferation, and progression. However, the apoptotic protease-activating factor 1 promoted cell to go to apoptosis.

A variety of glutathione-related proteins with high expression were identified, such as glutathione reductase (Fragment), glutathione S-transferase Mu 1, glutathione S-transferase Mu 2, glutathione S-transferase P, and glutathione S-transferase alpha-3. We thought that Fe_3_O_4_ NPs induced reactive oxygen species (ROS) to cause overexpression of glutathione-related proteins. After one day of Fe_3_O_4_ NPs treatment, a strong magnet was utilized to recover the residual NPs, but there were no NPs to be recycled. We considered that the NPs had transferred into ferric ions (Fe^3+^) and ferrous ions (Fe^2+^) and that endogenous hydrogen peroxide (H_2_O_2_) reacted with ferrous ions to compose hydroxide free radicals (Fenton reaction) [31, 32]. A variety of reports show that ROS effects are exhibited when NPs were treated [7, 14]. However, ferrous ion (Fe^2+^) is an initiator in the Fenton reaction, and ferrous ions will react with hydrogen peroxide to produce hydroxide free radicals.

### 3.5. Chaperone Proteins Overexpression and STRING Networks Establishment

We also characterized chaperone proteins such as heat shock proteins, serpin H1, protein disulfide-isomerase, endoplasmin, and endoplasmic reticulum resident protein.

**Figure 5:**
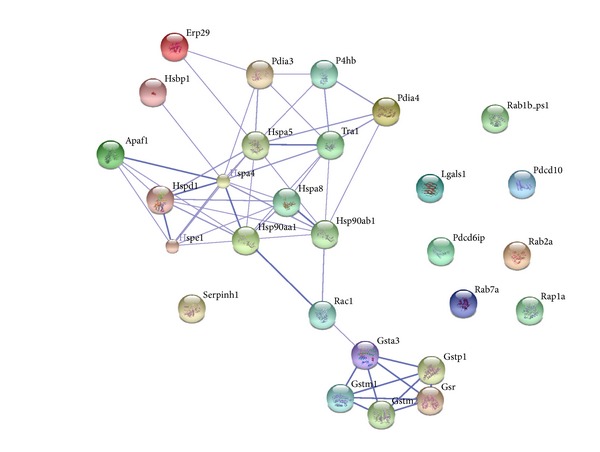
Schematic representation of the networks between cell death and apoptosis related proteins, ras-related proteins, glutathione related proteins, and chaperone proteins.

Eventually, we used the STRING (vision 9.1, http://string-db.org/) database to establish the interactions between chaperone proteins and related proteins to demonstrate the relationships between Fe_3_O_4_ NPs and the associated proteins [20]. The relationships between chaperone proteins, ras-related proteins, glutathione related proteins, and cell death and apoptosis related proteins are listed in Table 2 and illustrated by STRING in Figure 5. Heat shock proteins connected with each other and related ras-related C3 botulinum toxin substrate 1 and then combined to glutathione related proteins.

## 4. Conclusion

This is a pioneer experiment to show the cellular responses through Fe_3_O_4_ NPs treatment. We hypothesized an antagonistic effect in NRK-52E cell lines which is presented as cell death, apoptosis, and cancerization via programmed cell death protein and ras-related proteins; however there are protective mechanism proteins in NRK-52E such as chaperone proteins and glutathione related proteins. In future work, we shall generate or purchase Fe_3_O_4_ NPs in a smaller size to confirm the size effect of NPs, and we also need more evidence to prove the ROS effect induced via ferrous ions and to explain the tendency towards apoptosis or to avoid cell death.

## Supplementary Material

The supplementary information is the raw data generated all proteins in quantitative proteomics which are listed according to the D/H ratio priority.

## Figures and Tables

**Table 1:** The MS intensities of peptide GVDNTFADELVELSTALEHQEYITFLEDLK labeled H (*m*/*z* 1165.92) and labeled D (*m*/*z* 1168.60) in peak area and peak height.

*m*/*z*	Peak area	Peak height	Ratio of area	Ratio of height
1165.92	4084412	1240520	4.42	4.22
1168.60	18036323	5230797		

**Table 2:** Statistic classification of related proteins in NRK-52E cell lines with Fe_3_O_4_ NPs treatment.

UNIPROT accession^a^	UNIPROT accession number^a^	Protein identification	Molecular mass (kDa)^b^	Number of peptides^b^	Ratio^c^
Cell death and apoptosis related proteins					
APAF_RAT	Q9EPV5	Apoptotic protease-activating factor 1	146.1	2	780.9
PDC10_RAT	Q6NX65	Programmed cell death protein 10	25.0	2	233.2
LEG1_RAT	P11762	Galectin-1	15.5	5	1.75
PDC6I_RAT	Q9QZA2	Programmed cell death 6-interacting protein	99.2	n.d.	n.d.

Ras-related proteins					
RAB7L_RAT	Q63481	Ras-related protein Rab-7L1	23.7	2	3.2
RAB1A_RAT	Q6NYB7	Ras-related protein Rab-1A	23.4	13	2.3
RAB2A_RAT	P05712	Ras-related protein Rab-2A	24.1	3	1.6
RAC1_RAT	Q6RUV5	Ras-related C3 botulinum toxin substrate 1	22.4	2	1.2
RAP1A_RAT	P62836	Ras-related protein Rap-1A	21.8	n.d.	n.d.
RAB7A_RAT	P09527	Ras-related protein Rab-7a	24.3	n.d.	n.d.

Glutathione related proteins					
GSHR_RAT	P70619	Glutathione reductase (Fragment)	47.8	2	3.1
GSTM1_RAT	P04905	Glutathione S-transferase Mu 1	26.7	2	2.8
GSTM2_RAT	P08010	Glutathione S-transferase Mu 2	26.5	3	2.4
GSTP1_RAT	P04906	Glutathione S-transferase P	24.1	6	1.5
GSTA3_RAT	P04904	Glutathione S-transferase alpha-3	25.9	4	1.2

Chaperone proteins					
HSBP1_RAT	Q8K3X8	Heat shock factor-binding protein 1	8.7	2	2.8
HS90B_RAT	P34058	Heat shock protein HSP 90-beta	86.0	32	2.7
SERPH_RAT	P29457	Serpin H1	47.7	30	2.5
HS90A_RAT	P82995	Heat shock protein HSP 90-alpha	87.7	31	2.4
HSP74_RAT	O88600	Heat shock 70 kDa protein 4	97.0	2	2.2
HSP7C_RAT	P63018	Heat shock cognate 71 kDa protein	72.8	40	1.9
PDIA4_RAT	P38659	Protein disulfide-isomerase A4	75.1	9	1.8
CH60_RAT	P63039	60 kDa heat shock protein, mitochondrial	62.6	29	1.6
CH10_RAT	P26772	10 kDa heat shock protein, mitochondrial	11.2	3	1.6
GRP78_RAT	P06461	78 kDa glucose-regulated protein	74.4	19	1.6
ENPL_RAT	Q66HD0	Endoplasmin	95.4	17	1.6
PDIA1_RAT	P04785	Protein disulfide-isomerase	59.1	24	1.6
ERP29_RAT	P52555	Endoplasmic reticulum resident protein 29	29.3	3	1.6
PDIA6_RAT	Q63081	Protein disulfide-isomerase A6	49.6	4	1.5
PDIA3_RAT	P11598	Protein disulfide-isomerase A3	58.6	17	1.3

^a^Protein accession and protein accession number were received from the UNIPROT database available online: http://www.uniprot.org/uniprot (accessed on May 25, 2014).

^b^Molecular weight and number of protein peptides according to the Swiss-Prot database in the Mascot search engine.

^c^Ratio values of each protein according to protein list in the Mascot Distiller.
